# Miles Apart- The Needed Bridge Between Social Isolation And Transportation: The Case Of Central Minnesota

**DOI:** 10.1093/geroni/igaf122.3117

**Published:** 2025-12-31

**Authors:** Claudia Burgos Zuniga, Isaac Asirifi Boateng, Phyllis Greenberg

**Affiliations:** Saint Cloud State University, Saint Cloud, Minnesota, United States; St. Cloud State University, St. Cloud, Minnesota, United States; St. Cloud State University, St. Cloud, Minnesota, United States

## Abstract

Social relationships and its connection to transportation play a crucial role in the quality of life of many older adults (Black et al., 2015). While existing literature highlights the relationship between transportation access and social isolation, this poster focuses on the specific transportation challenges faced by older adults in Central Minnesota. Older adults in Central Minnesota expressed that lack of information (14%), availability of transportation (13%) and transportation costs (13%) were their most common barriers to accessing services (e.g., activities, wellness programs and IADL’s) (CMCOA, 2024). Additionally, some of the critical factors highlighted by research that can compromise older adult’s access to services and activities includes winter conditions, language barriers, technological gaps and safety concerns (Afnan et al., 2022; Dabelko-Schoeny et al.,2021). This poster proposes the following recommendations for consideration: · Promotion of age-friendly rural communities; · Direct (e.g., mobile, in-home) rural health care services; · Door to door transportation that is ADA compliant; · Liaison between school districts / colleges and senior centers in development of intergenerational exchange programs (e.g., skills and culture). By emphasizing localized solutions, this poster contributes to a deeper understanding of social isolation and offers actionable strategies to enhance the quality of life of older adults throughout the United States and internationally.

